# Restless Leg Syndrome in the Setting of Patients With End-Stage Renal Disease on Hemodialysis: A Literature Review

**DOI:** 10.7759/cureus.9965

**Published:** 2020-08-23

**Authors:** Srikala Kambampati, Shehnaz Wasim, Vishal Kukkar, Vanessa M Awad, Bilal Haider Malik

**Affiliations:** 1 Internal Medicine, California Institute of Behavioral Neurosciences & Psychology, Fairfield, USA; 2 Radiology, California Institute of Behavioral Neurosciences & Psychology, Fairfield, USA; 3 Internal Medicine/Family Medicine, California Institute of Behavioral Neurosciences & Psychology, Fairfield, USA

**Keywords:** restless leg syndrome, esrd, hemodialysis, uremic rls, quality of life

## Abstract

Restless Leg Syndrome (RLS), or Willis-Ekbom disease (WED), is an irresistible urge to move the legs, predominantly while resting, sitting, or sleeping, which disrupts sleep and impairs quality of life. RLS can occur secondary to uremia in chronic kidney disease (CKD) patients due to inadequate hemodialysis. Early diagnosis is essential to prevent muscular atrophy and to improve the quality of life of RLS patients, especially those with end-stage renal disease (ESRD). Cardiac mortality high in uremic RLS patients due to associated discomfort and lowering the duration of hemodialysis treatment. This review focuses on and discusses the diagnosis, treatment, and associated comorbid conditions of uremic RLS. Though the exact pathophysiology is unknown, altered transferrin expression in the choroid plexus, increased glutamate levels in the thalamus, decreased opioid receptors, dopamine system dysfunction, calcium/phosphate imbalance, and single nucleotide polymorphisms in the BTBD9 and MEIS1 genes are a few nonconfirmatory pathophysiological concepts for uremic RLS. Nonpharmacological options include lowering the temperature of dialysate by 1 degree C and home-based therapies like massages, warm/cold baths, and aerobic exercises. Pharmacological therapy like dopamine agonists ropinirole and pramipexole reduces the symptoms effectively. However, surgical options like parathyroidectomy and renal transplantation are stated as the best treatment options in patients suffering from uremic RLS.

## Introduction and background

Restless Leg Syndrome (RLS) is one of the most unrecognized chronic sensory-motor disorders in renal failure patients [1]. In the general adult US population, the prevalence of RLS is 5-10% [2]. The prevalence in patients undergoing hemodialysis therapy ranges from 8.8-83%, and the frequency of RLS in end-stage renal disease (ESRD) patients among different countries over the last 10 years is shown in Table 1 [3,4].

**Table 1:** Prevalence of restless leg syndrome (RLS)

Study	Year of publication	Country	Number of patients	Frequency of RLS (%)
Araujo SM et al., [5]	2010	Brazil	400	21.5%
Salman SM [6]	2011	Syrian	123	20.3%
Xiao CG et al., [7]	2012	China	375	13.3%
Irfan Haider et al. [8]	2014	Pakistan	250	64.8%

Restless leg syndrome, also called Willis-Ekbom disease (WED), is an uncomfortable urge to move the legs while resting, sitting, or sleeping, which disrupts the sleep. This unpleasant feeling dramatically affects the quality of life in these patients and can result in psychological disorders [9,10]. RLS/WED can be hereditary or acquired. Most patients have the primary idiopathic form of the disease, in which positive family history is present in nearly 40% of the cases, suggesting a genetic predisposition [11,12]. RLS predominantly affects legs or feet, but less commonly, it can involve arms. People describe symptoms as crawling, creeping, pulling, throbbing, aching, itching, and electric sensations. Renal failure and ESRD patients on hemodialysis constitute a large group of the population that are likely to be affected by RLS because of severe uremia. The presentation of RLS in the renal failure patients is highly variable, and the symptoms are intermittent and fluctuating, resulting in the late diagnosis in these patients.

Early diagnosis is crucial to improve the quality of life of RLS patients, especially those with ESRD. This review focus and discusses the diagnosis, treatment, and associated comorbid conditions of uremic RLS.

## Review

RLS is considered a sleep disorder and is more common in females than in males [13]. RLS can be primary or secondary. Primary or idiopathic RLS is mostly due to cerebral iron deficiency, dopamine system dysfunction, and increased glutamate levels. Secondary causes are pregnancy, amyloidosis, diabetes mellitus, magnesium and folate deficiencies, Lyme disease, and ESRD. Though the exact pathophysiology is still unknown, positive family history is present in 40% of cases (autosomal dominant inheritance pattern) [13]. Some studies have reported that there is an association of RLS with genetic factors like Chromosome 12q22-23, 14q13-21, and 9p24-22. Sometimes RLS presents early before age 45 due to underlying genetic associations. Therefore, there is an interaction between genes, gene modifiers, and environmental factors in RLS [14].

RLS occurs in patients with end-stage renal disease with severe uremia. Uremic RLS is associated with lower hemoglobin levels, transferrin saturation <20%, and inadequate response to epoetin alfa. Altered transferrin expression in the choroid plexus has been reported in autopsy studies. Iron is also necessary for neurologic dopamine recycling and is a crucial cofactor for the rate-limiting enzyme of dopamine synthesis (i.e., tyrosine hydroxylase) [13]. Other pathophysiology mechanisms described are increased glutamate levels in the thalamus, decreased opioid receptors, and serotonin-induced inhibition of dopamine transmission in patients taking antidepressant medication like selective serotonin reuptake inhibitors (SSRIs) and serotonin and norepinephrine reuptake inhibitors (SNRIs) [13]. Calcium/phosphate imbalance and single nucleotide polymorphisms in the BTBD9 and MEIS1 genes are significantly involved in the pathophysiology of uremic RLS [15].

Alcohol and caffeine usage

Studies show that women who drink alcohol have a higher chance of contracting this disease. The symptoms vary depending on the person. Caffeine, alcohol, antidepressants other than bupropion, antipsychotics, antiemetics, dopamine blockers, are centrally acting antihistamines. However, other studies show that cigarette smoking and coffee drinking is not related to RLS causes [16].

Metabolic derangements

According to research done at the Zaware laboratory [5], calcium or age is not associated with RLS. Araujo, on the other hand, found that RLS patients had lower hemoglobin, albumin, and alkaline phosphatase levels [11]. Santos et al. says that secondary hyperparathyroidism is associated with RLS exhibiting high phosphate and PTH [17]. Studies show that BMI is directly proportional to the association of uremic RLS due to the reduced number of dopamine receptors in obese patients' brains [15].

Chronic disease

Chronic kidney disease, diabetes, and oxidative stress are associated with uremic RLS [18]. Some studies say polyneuropathy is the highest risk factor of RLS in diabetic patients as it affects catecholaminergic systems by reducing the dopaminergic content, thus affecting the midbrain and striatum, which is crucial for RLS [7]. Tsai et al. found that longer duration of dialysis and a low HDL level is associated with RLS [19].

Diagnosis

Diagnosis of RLS is primarily clinical, based on an interview with the patient to answer the various questions based on the diagnostic criteria, as shown in Table 2 [20].

**Table 2:** National Sleep Foundation and RLS Foundation initial step questions

Table 2: National Sleep Foundation and the RLS Foundation questions to be asked by the clinician as an initial step.
Does a person have a strong desire to move his/her legs when he/she sit or lie down?
Does a person feel it impossible to resist his/her desire to move their legs?
Does a person ever use the words unpleasant, creepy-crawly, creeping, itching, pulling, or tugging to describe his/her symptoms?
Does a person's desire to move often occur when he/she is resting or sitting still?
Does a person moving his/her legs to make them feel better or slow down the symptoms?
Does a person ever awake, his/her partner in bed with their leg jerking movements
While a person is awake, does he/she ever had involuntary leg movements?
During the day, does a person able to concentrate appropriately or feel tired?
Does any of a person's family members have similar symptoms?
Do a person's symptoms are more predominant at night?
A recent visit to his/her doctor had not revealed any physical cause for their discomfort?

RLS does not have any specific diagnostic tests, therefore answering "Yes" to a majority of the questions and using International Restless Legs Syndrome Study Group (IRLSSG) standards to classify the disease into mild: RLS 0-10 points; Moderate: RLS 11-20 points; Severe: RLS 21-30 points; Very Severe RLS: 31-40 points helps in diagnosis (Table 3) [20].

**Table 3:** International RLS Severity Scale

Table 3: International RLS Severity Scale
How would a person rate the discomfort of RLS in his/her legs or arms? (0) None (1) Mild (2) Moderate (3) Severe (4) Very Severe
How would a person rate the need to move around because of RLS symptoms? (0) None (1) Mild (2) Moderate (3) Severe (4) Very Severe
How much relief of discomfort did a person get from moving around? (0) No RLS symptoms to be relieved (1) Either Complete or almost complete relief (2) Moderate relief (3) Slight relief (4) No relief
How severe was a person's sleep disturbance due to RLS symptoms? (0) None (1) Mild (2) Moderate (3) Severe (4) Very Severe
How severe was a person's tiredness or sleepiness due to RLS symptoms? (0) None (1) Mild (2) Moderate (3) Severe (4) Very Severe
How severe was a person RLS as a whole? (0) None (1) Mild (2) Moderate (3) Severe (4) Very Severe
How often did a person get the symptoms? (0) Never (1) Occasionally [<= 1h/24h] (2) Moderate [2-3 days/w] (3) Often [4-5 days/w] (4) Very often [6-7days/w]
When a person had the symptoms, how severe were they on average? (0) None (1) Mild [1hr/24hr] (2) Moderate [1-3hr/24hr] (3) Severe [3-8hr/24hr] (4) Very severe [>8 hour/24hr]
How severe was the impact of the symptoms on his/her ability to carry out daily affairs – for example, carrying out a satisfactory family, home, social, or work life? (0) None (1) Mild (2) Moderate (3) Severe (4) Very Severe
How severe was a person's mood disturbance due to RLS symptoms- for example, angry, depressed, sad, anxious, or irritable? (0) None (1) Mild (2) Moderate (3) Severe (4) Very Severe
Abbreviation: RLS, Restless legs syndrome. Scoring – mild: RLS 0-10 points; Moderate: RLS 11-20 points; Severe: RLS 21-30 points; Very Severe RLS: 31-40 points

A detailed family and social history, previous comorbid conditions, and medication history are essential to reach an appropriate diagnosis. Periodic leg movements during sleep (PLMS), akathisia due to autonomic failure of drug-induced peripheral vascular diseases, leg cramps at night, and painful legs along with moving toes are a few neurological conditions that should be differentiated from RLS, because they all are not relieved by walking, unlike RLS, which will temporarily reduce with movement.

Uremic RLS primarily affects the initiation of sleep and reduces sleep quality, affecting the quality of life (QoL) [21]. Uremic RLS patients' quality of life score was 25% lower than idiopathic RLS patients [22]. Twenty percent of the patients on hemodialysis reported premature discontinuation due to the development of RLS symptoms [23,24,25]. Besides, the legs twitch during the time of sleeplessness. They experience short sleep duration and insomnia during the daytime. Women are profoundly affected as compared to men who experience sleep during the day [26].

Giannaki et al. revealed the low QoL scores are primarily due to mental health and sleep-related aspects of uremic RLS [27]. It has been hypothesized that the muscle atrophy in RLS is because of the alterations of the growth hormone and insulin-like growth factors in these patients [27]. RLS and PLMS increase the risk of new/aggravation of existing cardiovascular disease in both uremic [28,29] and non-uremic [30,31,32] population. Increased blood pressure and heart rate during night time in idiopathic RLS [32] patients as well as the general population [33] due to increased sympathetic nervous system activity, leads to increased afterload resulting in left ventricular (LV) hypertrophy. According to Giannaki et al., uremic RLS with PLMS patients have increased LV internal diameters during diastole and more LV mass [34]. Uremic RLS in ESRD patients is associated with lower survival rate [24] and higher death [30].

Pharmacological treatment

Dopamine Pathways

Many treatments work with dopamine agonists being the first choice. It is known to have a first and immediate action. Non-ergot dopamine agonists like ropinirole and pramipexole are used primarily for the treatment of RLS [35]. Ropinirole was more effective than levodopa in treating uremic RLS by reducing 75% of the symptom [6]. Pramipexole, taken two hours before sleep, was shown to be effective in improving periodic limb movements and reducing uremic RLS symptoms by 70% [35]. Dopamine activates the receptors within the brain, regulating the movement and moods of the RLS patient. However, large amounts of electrolyte shifts affect dopamine synthesis by directly or indirectly influencing both the cellular and subcellular processes during dialysis. Studies say this could be one of the reasons to have increased frequency of RLS in hemodialysis patients compared to peritoneal and non-hemodialysis chronic renal failure patients [36]. Generally, these medications show that empowering brings about dialysis patients. However, the proof base stays little and more investigations are required to take into consideration more grounded suggestions for their utilization.

Gabapentinoids

Gabapentin and pregabalin are believed to have a curative effect on RLS. Pregabalin and gabapentin are analogs to the inhibitory neurotransmitter. Their principal system of activity is through the presynaptic official to the alpha2-delta subunit of voltage-gated calcium channels, leading to the decrease of synapse discharge, weakening neuronal hyperexcitability, and irregular synchronization. These investigations state that gabapentinoid is a protected and successful treatment for the syndrome in patients. However, utilization is constrained to some degree by an absence of bigger randomized controlled preliminaries [37]. Therefore a kidney transplant seems to be the best treatment for uremic RLS.

Non-pharmacological treatment

Sakkas et al. found that cooling of the dialysate solution from 37 to 36 degrees C effectively reduced the motor and sensory symptoms of uremic RLS and is considered a safe nonpharmacological option to overcome the symptoms as well as to improve the restlessness [38].

Home-Based Therapies

Results state that RLS has no immediate medical cure, but the home-based treatment options like massage, warm baths, warm/cold compresses, relaxation techniques, changes to sleep environment, and exercise are effective in alleviating RLS symptoms.

Studies state that when the body is at rest, there is a likelihood of a high rise in the levels of RLS. Aerobic exercise is identified to decrease the sovereignty of the diseases. However, sleeping parameters are not assessed. Therefore, this indicated the patients should take a lower dose of dopamine agnostic and exercise regularly with home-based care therapies known to help [37].

Comorbid Conditions Treatment

Most antidepressants worsen RLS symptoms, but studies show that bupropion does not exacerbate RLS symptoms and is the drug of choice to treat depression in uremic RLS [39]. Studies are yet to prove the beneficiary effect of parathyroidectomy in treating secondary hyperparathyroidism and high serum phosphate levels associated with uremic RLS [17].

**Figure 1:**
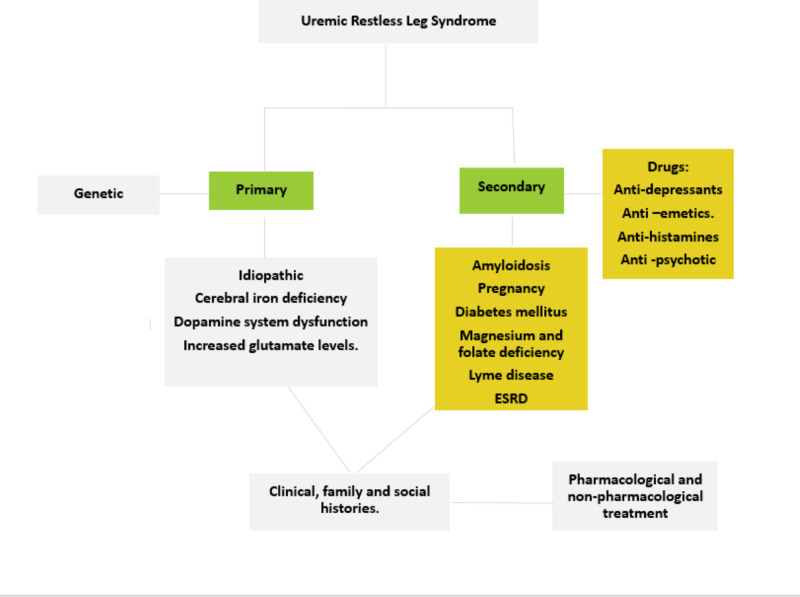
Review of uremic restless leg syndrome (RLS)

The overall review is seen in Figure 1. The quality assessment of this article has not done. We call upon further studies to find the exact pathophysiology, as very little is known. Encourage physicians to use methods for early diagnosis to prevent morbidity and mortality. In addition to this, more research should be done to improve the pharmacological options of uremic RLS and not necessarily rely on kidney transplant as the only possible treatment available for the disease.

## Conclusions

Restless Leg Syndrome is a common unrecognized disorder in ESRD patients on hemodialysis. Meticulous history taking and observation are needed to clinch the diagnosis. Uremic RLS associated with PLMS has a worse prognosis. Effective hemodialysis and renal transplantation is the treatment of choice. Of the available drugs, ropinirole and pramipexole are effective in reducing symptoms. Aerobic exercises and lower extremity strength training exercises are nonpharmacological options to reduce the symptoms and to improve the quality of life. A breakthrough in the cure of RLS is dependent on how vast is the disease research to curb the problem. More research is needed to find dietary variations and races associated with RLS. Researchers and scientists can come up with ways to find a more advanced treatment with fewer side effects to the patients, as witnessed with the already available treatment where cases of dropout are prevalent due to the side effects of the medicine.
